# On Monomania

**Published:** 1849-08

**Authors:** 


*On Monomania.* By M. Baillarger.—The author has devoted a clinical lecture at the Saltpêtrière to the consideration of this subject He defines monomania to be a partial insanity, resulting from an exciting passion; differing in this respect from melancholia, which is produced by a depressing passion or emotion. M. Baillarger accounts for the opinion of Foville and some other authors, as to the rarity of this disorder, by the fact, that many persons laboring under it may succeed in concealing their state from their friends and physician, until the mental derangement has become so great as to preclude the affected individuals being longer regarded as simple monomaniacs. In proof of the general correctness of this observation, he instances two cases:—The first, that of a soldier, who contended for more than twenty years against a constant and powerful desire to murder certain of his relations. Before this propensity became so irresistible, he was forced to acquaint his friends with the power it exercised over him, in order that proper restraint might prevent him from doing the mischief to which he was impelled. The second case is that of a lady, whose hallucination was of a more amiable and harmless character; consisting simply in an overweening concern about the possibility of her regarding her husband with the proper degree of conjugal devotion. This state of mind had existed for three years, to the great distress of the patient, but unattended by any further symptoms of insanity. Hallucinations of this nature are called by the author, and other French writers, "fixed ideas;" they are, for the most part, carefully concealed from notice by the subjects who are rendered so unhappy by their possession. Monomaniacs, therefore, according to the author's definition, are rarely found confined in asylums; they move unchallenged in society, and it is only when very particular attention is directed to the inquiry, that anything like an adequate idea of the number of persons so affected can be obtained.

*Causes of Monomania.*—An extreme degree of mental sensibility or irritability is supposed by the author to act most frequently as the *predisposing* cause of monomania, accompanied in several, though by no means in all instances, by a limited degree of mental endowment. Certain events produce a more or less permanent impression on the minds of some individuals, which, on those of others, exercise a very slight or transitory influence;—such individuals are precisely those in whom we may expect the "fixed ideas" of monomania to become developed. This peculiar impressibility of the mind is more common and better marked in women than in men; and in the former, more particularly, during the existence of certain physiological states proper to their sex. The *occasional* or *exciting* causes of monomania are various; among these, lively mental emotions, especially violent grief, occupy a prominent place. The author quotes several cases in illustration of this fact; of these, the following is one of the most interesting:—Augusta Strohen, when a young girl, witnessed the execution of a criminal at Dresden. The general interest felt in his fate, and the important part he played in the tragedy of the execution, made a deep and lasting impression on her mind; and the desire thus excited, of occupying a similarly conspicuous position in the regards of the public, became at last sufficiently strong to drive her for its gratification to the commission of murder. Irritation is also a common cause of monomaniacal insanity; and to this cause may be traced many of the instances on record of homicidal and suicidal epidemics. This fact illustrates the mischievous tendency of those newspaper paragraphs devoted to stories of murders and executions. A striking dream occasionally acts as the exciting cause of monomania; in this case the "fixed idea" corresponds with the most remarkable idea in the dream. A curious circumstance in the history of typhus fever, is the occasional determination of the accompanying delirium, after the febrile excitement has become somewhat mitigated, in one particular and exclusive direction. M. Louis has recorded some cases of monomania originating in this way; in which, however, the aberration was only temporary, having been corrected after convalescence was considerably advanced. Similar effects sometimes follow the delirium of the "cerebral fevers" of women recently delivered; and it is remarkable, that the insanity which remains after such attacks, which may be monomaniacal or more general in its character, is more common among women of the higher than of the lower classes,—obviously from the greater mental susceptibility encouraged by the habits and education of the former.—*Lond. Monthly Journal, from Gaz. des Hôpitaux.*

